# Validity and reliability of the Chinese version of the Patient-Reported Outcomes Measurement Information System adult profile-57 (PROMIS-57)

**DOI:** 10.1186/s12955-022-01997-9

**Published:** 2022-06-15

**Authors:** Tingting Cai, Fulei Wu, Qingmei Huang, Chunfang Yu, Yang Yang, Feixia Ni, Changrong Yuan

**Affiliations:** 1 School of Nursing, Fudan University, 305 Fenglin Road, Shanghai, 200032 China; 2 Department of Hematology, The Second Affiliated Hospital of Guilin Medical University, Guangxi, China; 3 Department of Nursing, Fudan University Shanghai Cancer Center, Shanghai, China

**Keywords:** Breast cancer, Evaluation, Patient-reported outcomes, HRQoL

## Abstract

**Background:**

The PROMIS-57 is a commonly used self-reported instrument to solve the lack of generalizable and universal measures required to evaluate common symptoms and functions from patients’ perspectives. This study aimed to translate the PROMIS-57 into Chinese and psychometrically test the translated instrument on patients with breast cancer.

**Methods:**

Translation, cross‑cultural adaptation, and psychometric evaluation of the instrument were performed from June 2020 to June 2021. Eligible patients were recruited and completed the PROMIS-57, Functional Assessment of Cancer Therapy-Breast (FACT-B), and a sociodemographic questionnaire.

**Results:**

Data from 602 patients with a mean age of 48.83 years were analyzed. Most domains in the PROMIS-57 showed an absence of floor and ceiling effects. Multi-trait scaling analysis demonstrated acceptable convergent and discriminant validity. The correlations between the PROMIS-57 scores and the selected FACT-B scores supported the criterion validity via the Pearson correlation test. Measurement invariance was supported by the absence of differential item functioning for most items. Cronbach’s α of the domains ranged from 0.85–0.95. The unidimensional factor structure of all domains was supported using confirmatory factor analyses. Additionally, most items showed acceptable item information curves and item characteristics curve matrices.

**Conclusion:**

The Chinese version of the PROMIS-57 was found to be a reliable and valid tool for assessing common symptoms and functions among patients with breast cancer.

## Background

Patients with breast cancer experience multiple psychological, physical, and social dysfunctions owing to the cancer and associated medical treatment [1]. Frequent and accurate assessment of symptoms and functions is an essential element for the care of patients with cancer, in which patient-reported outcome (PRO) measures have been a gold standard to quantify patients’ subjective experience [2]. Valid and reliable PRO measures are vital for patient-centered care among those with breast cancer, as they play an important role in enhancing patient-nurse communication and assisting nurses in systematically monitoring and managing patients’ symptoms [1, 3]. In addition, such assessments enable the prediction of the symptom trajectory for this vulnerable sample [4]. There is a great demand for evaluations of PROs across a broad range of health domains in patients with breast cancer.

The Patient-Reported Outcomes Measurement Information System (PROMIS) is a program initiated by the National Institutes of Health, aiming to promote the use of valid and generalizable instruments across different health conditions. Under the framework of the PROMIS, more than 100 domains were identified to be relevant for health-related quality of life (HRQoL) for most health contexts [5]. Many HRQoL instruments are developed using classical test theory (CTT). Item response theory (IRT) analysis is more capable of guiding item selection [5]. Brief items with patient-centered content, IRT, or other psychometric methods will contribute to assessing HRQoL domains in routine clinical care. The PROMIS-57, a self-reported instrument, was developed to solve the lack of generalizable and universal measures required to evaluate common symptoms and functions from patients’ perspectives [6, 7]. All the informative items of the PROMIS-57 were selected based on a literature review, IRT analysis, and rounds of expert review of psychometric evaluation findings from PROMIS datasets. The PROMIS-57 has a standardized scoring system, and covers core patient-reported symptoms and functional domains for patients with chronic diseases.

Given the brevity, breadth, and strong psychometric properties of the original English version of the PROMIS-57, the instrument has been translated into more than 40 cross-cultural adapted versions with satisfactory psychometric properties across different clinical conditions, including patients with cancer [5]. The availability of translated versions of the PROMIS-57 might contribute to international comparisons of health domains and enable sharing of effective interventions. Considering that a patient-reported outcome measure is needed to facilitate the assessment of common symptoms and functioning in patients with breast cancer, the PROMIS-57 is an optimal instrument to satisfy the clinical and research use in this population [5].

China has a large population of patients with breast cancer, with the mean age at which the cancer was diagnosed being significantly younger than that of their Western counterparts [8]. Young patients are more vulnerable to cancer [8]. However, a simplified Chinese version of the PROMIS-57 is not available. Considering the importance of assessing physical, social, and mental health issues in patients with breast cancer, the availability of the PROMIS-57 in China would not only have clinical use but also allow for international comparisons with the patients in other countries. Therefore, the current study addressed the need for a simplified Chinese version of the PROMIS-57 by translating the original English version and performing a psychometric evaluation in a sample of patients with breast cancer in mainland China.

## Methods

### Study design

A cross-sectional research design and convenience sampling were utilized, adhering to the STROBE statement. First, we translated and cross‑culturally adapted the PROMIS-57. Then, we performed a psychometric evaluation of the Chinese version of the instrument among patients with breast cancer following the practices recommended by Consensus-based standards for the selection of health measurement instruments (COSMIN). The phase two data were collected between June 2020 and June 2021.

#### Phase 1: translation and cross-cultural adaptation

**Fig. 1:**
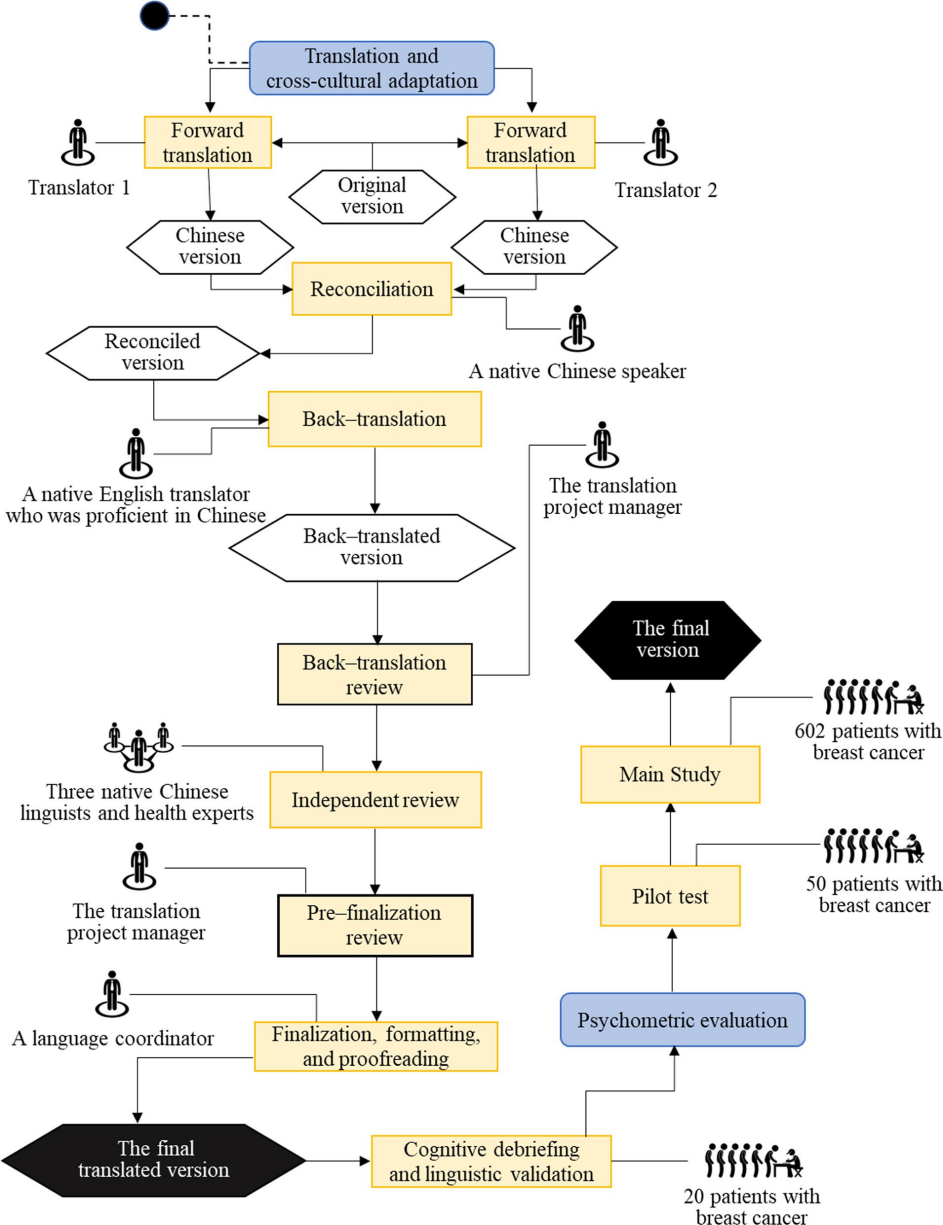
Flow of the translation and cross‑cultural adaptation

The Functional Assessment of Chronic Illness Therapy (FACIT) method—recommended by the PROMIS^®^ Health Organization—was applied to develop the simplified Chinese version of the PROMIS-57 [9]. All the included translators involved in the procedure had a master’s degree or above. In accordance with the translation guideline, the translation procedure incorporated forward translations, reconciliation, back-translation, back-translation review, independent review, pre-finalization review, finalization, formatting and proofreading, harmonization, cognitive debriefing, and linguistic validation [10] (Fig. 1):

1. Forward translation. Two translators who were bilingual native Chinese speakers translated the PROMIS-57 into the Chinese version independently.
2. Reconciliation. A native Chinese speaker compared the two translated versions and reconciled the discrepancies or made necessary changes to optimize the translations. Afterward, a reconciled version that included the best of the former translations was created.
3. Back-translation. The reconciled version of the instrument was back-translated by a translator who was a native English-speaking scholar and was fluent in Mandarin Chinese. Additionally, the translator had not seen nor had any knowledge of the PROMIS-57.
4. Back-translation review. The translation project manager, who had extensive experience translating and cross‑culturally adapting PROMIS measures, compared the items and the equivalence in the original and the back-translated versions, and made suggestions regarding any possible changes to ensure the equivalence in meaning.
5. Independent review. Three healthcare professionals who were native Chinese speakers reviewed all translated versions and determined the most appropriate one for each item or provided alternate translations.
6. Pre-finalization review. A thorough review was performed by the translation project manager regarding all the previous translations in addition to the comments of the translators and made necessary changes to improve the translations.
7. Finalization. A language coordinator determined the final translation based on the translation project manager’s comments and the preceding information and performed language modification.
8. Formatting and proofreading. Spelling and grammatical issues were checked and reconciled for the translated version by two proofreaders independently.
9. Harmonization. The equivalence of the decided translation was reviewed and revised by native English speakers who had experience developing PROMIS measures.
10. Cognitive debriefing and linguistic validation. A cognitive review was performed with 20 individuals with breast cancer for the translated PROMIS-57, aiming to verify the comprehensibility, understandability, and equivalence of the instructions, items, and response options. Modifications were accordingly made for the final translation of the instrument to ensure that the expression of items and instructions was equivalent to the original English version. The instrument was subsequently pilot-tested in 50 patients with breast cancer.

#### Phase 2: psychometric evaluation

##### Setting and sample

The translated measure was validated in participants from two tertiary hospitals in Shanghai, China. Patients were recruited from the department of breast surgery or oncology surgery. To be eligible, patients needed to be aged 18 years or older, with a diagnosis of breast cancer, and able to read and speak Mandarin. Patients with cognitive deficits or serious medical conditions were excluded.

##### Measures

*Demographics* Demographic characteristics, including age, marital status, number of children, education level, employment status, and monthly family income, were self-reported by patients. Clinical data such as cancer stage, surgery type, and therapeutic regimen were also collected from patients’ medical records by the research staff.

*PROMIS-57* The PROMIS-57 version 2.1 is a 57-item instrument that assesses seven domains: anxiety, depression, physical function, fatigue, sleep disturbance, ability to participate in social roles and activities, and pain interference and intensity. Except for a single item for pain intensity, all domains include eight questions and are responded to with a five-point Likert scale [11]. The pain intensity item is answered with a 0–10 numeric rating scale ranging from 0 (*without pain*) to 10 (*worst pain imaginable*). Item scores in each domain were summed and transformed into T-scores metric: values of 50 (SD = 10) indicate the mean of the U.S. general population (http://www.healthmeasures.net). A higher score indicates a greater magnitude of the concept being measured [11].

*Functional Assessment of Cancer Therapy-Breast (FACT-B)* The FACT-B instrument was employed to evaluate the construct validity of the PROMIS-57. FACT-B is a validated and frequently used instrument to measure the quality of life in Chinese patients with breast cancer [12]. It contains 36 items across five domains: physical, social/family, emotional, functional well-being, and a breast cancer-specific subscale. A summated five-point Likert scale is applied to report respondents’ diverse levels of health domains, with responses ranging from 0 (*not at all*) to 4 (*very much*). The numbered responses in all five subscales were then added together to create a sum score ranging from 0 to 144. Higher scores indicate better quality of life. Cronbach’s α was 0.91 in this study.

### Data collection

Ethical approval was approved by the Institutional Review Boards of Fudan University Cancer Hospital (no 1810192-22) and Fudan University Zhongshan Hospital (no 2020-076R). Patients were enrolled during hospitalization by the research staff, who had received training regarding the study process to ensure the standardization of the data collection. Participants were approached after being screened for eligibility. Participants received written information about the study’s aim and procedure before the start of the study. In addition, they were informed about the anonymity of their information and that all responses would be kept confidential and used only for research purposes.

### Data analysis

All data were analyzed with SPSS version 21.0, Mplus 7.4, and MultiLog 7.03. Statistical significance was set at p < 0.05 for all analyses.

Descriptive statistics were calculated for sample characteristics and study variables, in which continuous variables were analyzed by means and standard deviations, and categorical variables were described by counts and percentages. The PROMIS-57 raw scores were transformed into T-scores based on the PROMIS guidelines (http://www.healthmeasures.net). For all domains, percentages of the responses greater than 15% of all participants with the highest or lowest scores indicated ceiling or floor effects, respectively [13].

Pearson correlation tests were performed to assess the convergent validity and discriminant validity. The correlation coefficient between each item and their own domains were hypothesized to be 0.40 or greater between the item and its domains. On the other hand, discriminant validity was examined by the correlation coefficient between each item and other domains. A higher correlation for each item with its own domain than that with other domains were indicators of satisfied convergent validity and discriminant validity [14].

Criterion validity was evaluated by computing the correlations of the PROMIS-57 domains and other domains of similar constructs in the FACT-B. Pearson correlation coefficients > 0.50 were considered strong correlation, 0.30–0.50 indicated moderate correlation, and < 0.30 indicated weak correlation [9]. Measurement invariance of the PROMIS-57 was examined using differential item functioning (DIF). DIF indicates whether respondents from various groups perform differently on the instrument even though they have the same degree of the studied trait [9]. DIF was calculated for the instrument regarding age and education by using the model of multiple-indicator multiple-cause. Internal consistency were assessed with Cronbach’s α coefficients, with values greater than 0.70 being acceptable [15].

IRT-based psychometric evaluation was performed with unidimensionality, item information curves, and item characteristics curve (ICC) matrices. Each domain in the PROMIS-57 was expected to have a one-fact structure to be consistent with the hypothesis of IRT for items in a scale. A confirmatory factor analysis (CFA) confirmed the unidimensionality structure of each domain via the MLM CFA estimator method. The good model fit was defined using the comparative fit index (CFI, values > 0.95), Tucker-Lewis Index (TLI, values > 0.95), root mean square error of approximation (RMSEA, values < 0.06), and standard root mean square residual (SRMR < 0.08) [16, 17]. ICC demonstrated the correlation between the ability of participants and the probability of correct reflection of items [18]. The graded response model created an ICC for each option of each item in the scale. An ideal ICC curve should show the monotonic variation of the first and fifth lines, with a normal distribution curve in the middle [18]. Regarding item information curves, the larger the values, the smaller the measurement standard error [19]. An ideal information curve should be in the shape of a “plateau”; that is, a higher amount of information can be obtained from a wider range [19].

## Results

### Cross-cultural adaptation

A total of 20 patients with breast cancer participated in the cognitive debriefing in terms of instructions, items and item options of the PROMIS-57. The average age of the patients was 58.30 years. Younger and more educated patients needed less help completing the questionnaire than older and less educated patients. Most patients did not have trouble understanding the items. However, some items were found to have cultural differences. Thus, a linguistic validation was performed for the translation to ensure that the instrument was appropriate in the Chinese cultural context. Overall, the results demonstrated that the translations of instructions, items, and response options in the PROMIS-57 maintained the linguistic equivalence with the original English versions. With a few exceptions, most items were easy to comprehend and culturally appropriate. Key changes included cultural differences in some words, such as “vacuuming,” which is not common in China. Therefore, the word was changed to “cleaning” in the Chinese version. Some words such as “running errands” were not easy to translate. Alternative translations with expansions were provided to reflect the closest meaning. The revised translation was subsequently pilot-tested and was validated on 50 patients with breast cancer, with satisfactory results; thus, it was ready for psychometric testing.

### Sample characteristics

Six hundred seventy patients with breast cancer were eligible after screening. Thirty-six declined participations owing to other time commitments. Of the remaining 634 participants, 32 were excluded because of too many missing items (more than four items were not answered), or that they were deemed to have made random responses (chose the same options for all items). Thus, data from 602 patients with a mean age of 48.83 years were analyzed (range = 24–79 years). As shown in Table 1, most patients were married (93.0%), with child or children (97%), had a secondary school education (30.2%), were unemployed (32.2%), had a monthly family income < ¥5000 (48.5%), and had employee insurance (74.1%). Regarding the cancer-related characteristics, most had stage III breast cancer (39.5%), received breast-conserving surgery (38.9%), and underwent a combined treatment of surgery and chemotherapy (33.2%).

**Table 1:** Sample characteristics of the study sample (N = 602)

Variables	n (%)
*Age in years* *(Mean ± SD)*	48.83 ± 9.74
*Marital status*	
Single	12 (2.00)
Married	560 (93.0)
Divorced	17 (2.8)
Widowed	13 (2.2)
*Children*	
0	18 (3.0)
1	383 (63.6)
2	167 (27.7)
≥ 3	34 (5.7)
*Education level*	
Primary school or below	131 (21.8)
Secondary school	182 (30.2)
High school	113 (18.8)
University or above	176 (29.2)
*Employment status*	
Employed	124 (20.6)
Medical leave	119 (19.8)
Unemployed	194 (32.2)
Retired	165 (27.4)
*Monthly family income*	
≤ ¥5000 (USD $770)	292 (48.5)
¥5001–10,000	202 (33.6)
> ¥10,001 (USD $1543)	108 (17.9)
*Medical insurance*	
Free medical insurance	6 (1.0)
Employee health insurance	446 (74.1)
Rural health insurance	113 (18.8)
Without health insurance	37 (6.1)
*Cancer stage*	
I	60 (10.0)
II	168 (27.9)
III	238 (39.5)
IV	66 (11.0)
Do not know yet	70 (11.6)
*Surgery type*	
Modified radical mastectomy	158 (26.2)
Breast-conserving surgery	234 (38.9)
Simple mastectomy	33 (5.5)
Other operations	177 (29.4)
Therapeutic regimen	
*Surgical treatment*	31 (5.1)
Surgery + chemotherapy	200 (33.2)
Surgery + radiotherapy	18 (3.0)
Surgery + chemotherapy + radiotherapy	110 (18.3)
Surgery + chemotherapy + radiotherapy + endocrine therapy	158 (26.2)
Combination of therapies	85 (14.2)

¥ Chinese yuan, *USD* United States dollar

### Descriptive statistics, ceiling, and floor statistics

As shown in Table 2, mean T-scores of physical function (41.80 ± 9.04) and social health (42.99 ± 10.55) domain scores were significantly lower than the reference level according to the PROMIS guidelines (http://www.healthmeasures.net). No evidence of floor or ceiling effects were identified in any of the domains in the PROMIS-57 (range: 0.17%–13.48%), except for the anxiety domain. The ceiling effect was found in the anxiety domain (16.3% of the participants) (Table 2).

**Table 2:** T-scores, floor and ceiling effects of the PROMIS-57

Domain	Mean	SD	Floor, n (%)	Ceiling, n (%)
Physical function	41.80	9.04	2.83	4.33
Anxiety	52.48	10.08	0.17	16.30
Depression	52.02	8.87	0.17	13.48
Fatigue	49.65	6.32	0.83	4.83
Sleep disturbance	49.36	7.92	1.33	4.16
Ability to participate in social roles and activities	42.99	10.55	5.32	11.98
Pain interference	52.66	8.73	1.33	9.32

### Convergent validity and discriminant validity

The correlations between each item with its domains were all greater than 0.40 (range: 0.69–0.93, p < 0.05) and were higher than with other domains, showing acceptable convergent validity and discriminant validity (Table 3).

**Table 3:** Pearson correlation coefficients for the construct validity of the PROMIS-57

Domain	Pearson correlation coefficients						
	Physical function	Anxiety	Depression	Fatigue	Sleep disturbance	Ability to participate in social roles and activities	Pain interference
Physical function	0.81–0.90	0.23–0.33	0.12–0.25	0.16–0.21	0.27–0.37	0.37–0.45	0.13–0.26
Anxiety	0.17–0.23	0.77–0.91	0.54–0.68	0.36–0.48	0.36–0.46	0.13–0.26	0.27–0.37
Depression	0.14–0.24	0.61–0.77	0.81–0.91	0.55–0.67	0.37–0.44	0.12–0.20	0.33–0.46
Fatigue	0.17–0.20	0.31–0.42	0.46–0.51	0.69–0.84	0.37–0.51	0.19–0.29	0.42–0.54
Sleep disturbance	0.21–0.40	0.30–0.39	0.27–0.43	0.27–0.42	0.70–0.81	0.14–0.27	0.20–0.33
Ability to participate in social roles and activities	0.40–0.49	0.20–0.23	0.12–0.21	0.21–0.32	0.28–0.38	0.88–0.93	0.15–0.28
Pain interference	0.12–0.21	0.31–0.37	0.34–0.43	0.36–0.47	0.23–0.38	0.20–0.29	0.75–0.88

### Criterion validity

The correlations between PROMIS-57 item scores with the corresponding domains coefficients in the FACT-B ranged from 0.32–0.56 (p < 0.05), showing satisfactory construct validity (Table 4).

**Table 4:** Pearson correlation coefficients for the construct validity of the PROMIS-57

Domain	Physical well-being	Social/family well-being	Emotional well-being	Functional well-being
Physical function	0.56^*^			
Anxiety			0.43^*^	
Depression			0.39^*^	
Fatigue				0.40^*^
Sleep disturbance				0.32^*^
Ability to participate in social roles and activities		0.51^*^		
Pain interference				0.36^*^

^*^p < 0.05

### Measurement invariance

The multiple-indicator multiple-cause model indicated that no significant DIF existed in most of the PROMIS-57 items in patients with different ages and education backgrounds, except one item in the anxiety and depression domain, respectively, suggesting that the instrument provided unbiased results in this population overall. We did not investigate DIF in gender, as we had only recruited female patients in this study.

### Reliability

A total of 50 patients with breast cancer were included for the pilot study of the PROMIS-57. The Cronbach’s α coefficient of each short form was 0.87–0.93, indicating acceptable reliability across all domains. In the psychometric evaluation of the PROMIS-57, Cronbach’s α coefficients for all PROMIS-57 domains were above the threshold of 0.70, ranging from 0.85 (fatigue) to 0.95 (physical function, anxiety). In addition, the split-half coefficient ranged from 0.78 (fatigue) to 0.94 (social health domain), indicating sufficient reliability (Table 5).

**Table 5:** Reliability of the PROMIS-57

Domain	Cronbach’s α	Split-half coefficient
Physical function	0.95	0.87
Anxiety	0.95	0.92
Depression	0.91	0.93
Fatigue	0.85	0.78
Sleep disturbance	0.87	0.81
Ability to participate in social roles and activities	0.93	0.94
Pain interference	0.92	0.91

### Unidimensionality

**Fig. 2:**
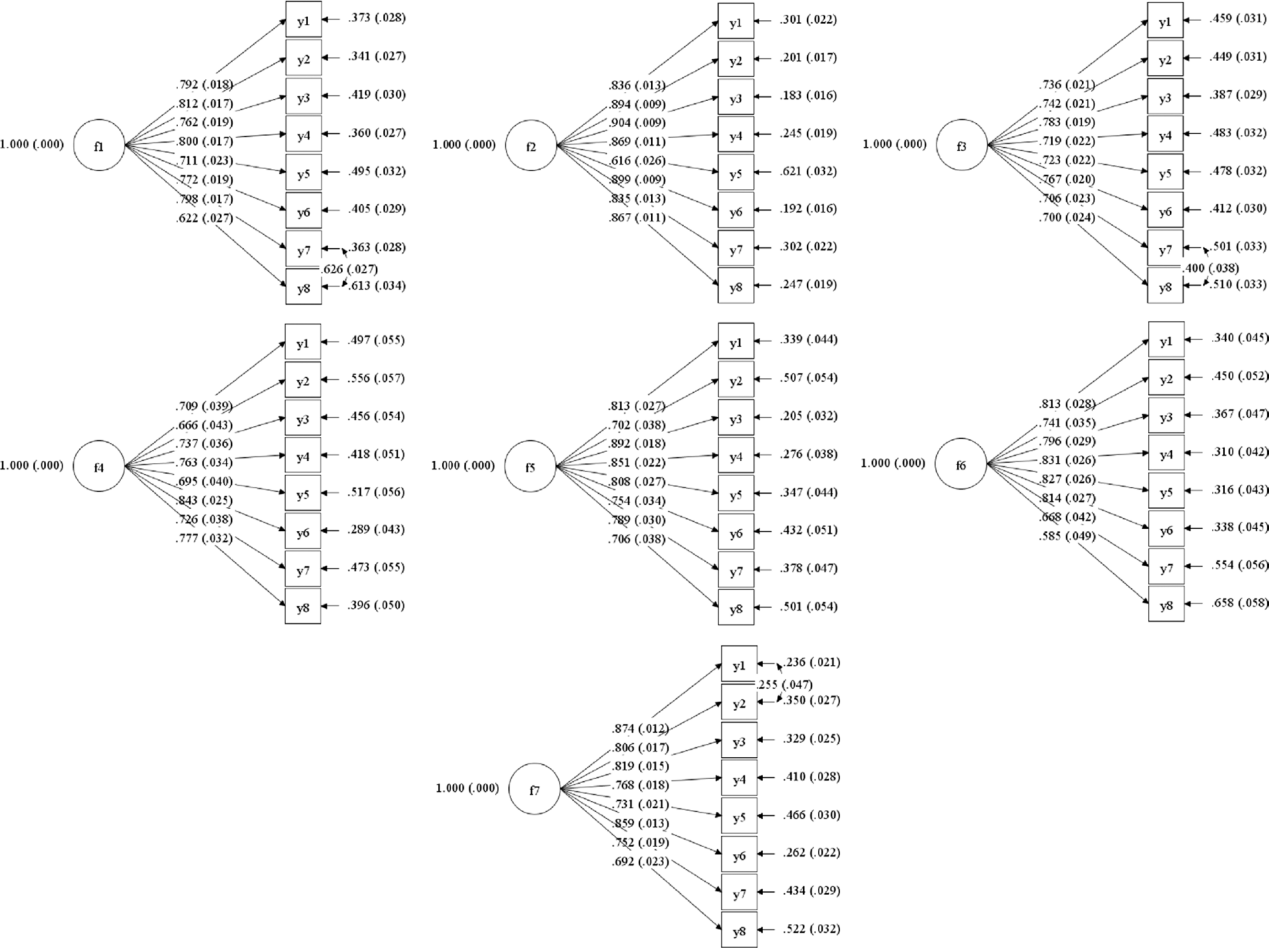
CFA models for each PROMIS-57 domain (f1–f7: anxiety, depression, physical function, fatigue, sleep disturbance, ability to participate in social roles and activities, and pain interference, respectively)

A one-factor structure was supported in all CFA models of each PROMIS-57 domain, with factor loadings ranging from 0.579–0.959 (Table 6; Fig. 2). Therefore, all domains showed sufficient model fit for unidimensionality structure.

**Table 6:** Model fit indices of confirmatory factor analysis for each PROMIS-57 domain

Domain	χ^2^	df	CFI	TLI	RMSEA	SRMR
Physical function	67.88	28	0.953	0.961	0.042	0.027
Anxiety	51.89	20	0.969	0.957	0.039	0.024
Depression	71.17	31	0.954	0.953	0.056	0.031
Fatigue	49.02	20	0.992	0.989	0.047	0.021
Sleep disturbance	85.03	28	0.979	0.986	0.046	0.019
Ability to participate in social roles and activities	46.05	20	0.971	0.959	0.049	0.033
Pain interference	58.34	25	0.988	0.983	0.052	0.014

*CFI* comparative fit index, *TLI* Tucker-Lewis Index, *RMSEA* root mean square error of approximation, *SRMR* standard root mean square residual

### Item information curves and item characteristics curves

**Fig. 3:**
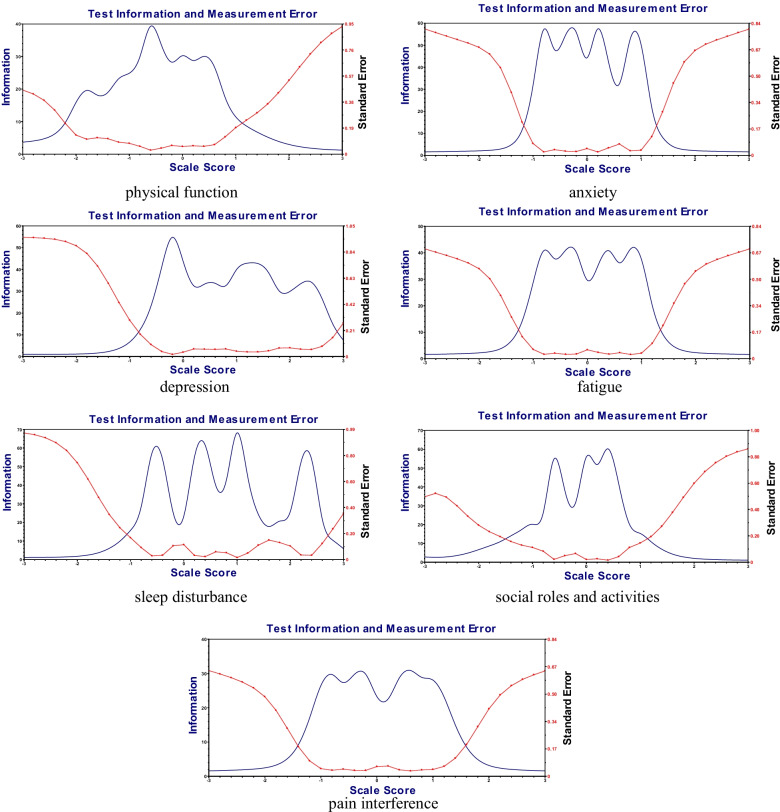
Item information curve for each PROMIS-57 domain

The results of item information curves in each domain demonstrated that all items scored high on information, well above the cut-off of 30, indicating that all items were within the highly reliable range of measurement for each domain (Fig. 3).

**Fig. 4:**
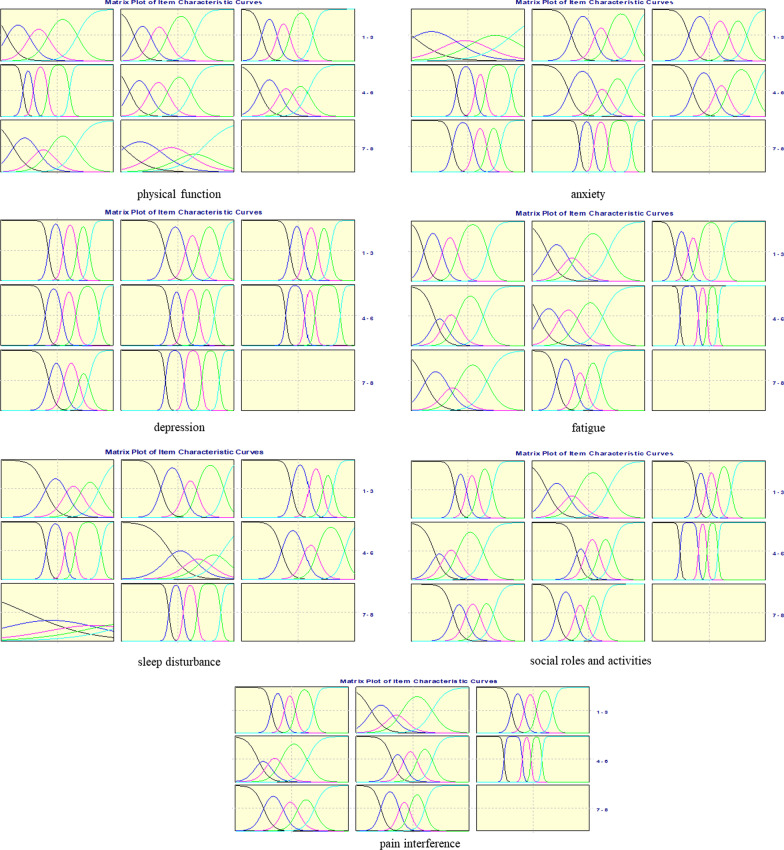
Item characteristics curve matrix for each PROMIS-57 domain

As can be seen from Fig. 4, with few exceptions, the ICC matrix for each item of the PROMIS-57 generally conformed to the hypothesis, in which the first and fifth ICC curves generally showed monotonous changes, while the middle curve presented a normal distribution, except item 7 for sleep disturbance.

## Discussion

This study was the first to translate the PROMIS-57 into a simplified Chinese version and test its psychometric properties in patients with breast cancer. We followed the recommended steps of the FACIT translation methods. The results showed that most items were simple and easy to understand, while a few items had cultural differences. Based on the participants’ feedback, the research team selected the most appropriate translation after considering the context and cultural differences, and the translated instrument was understandable and had cultural equivalence in patients with breast cancer.

The T-scores of the PROMIS-57 indicated that physical function and social health domain were lower than those of the reference group. The participants’ physical function score was the lowest of the seven health domains, suggesting that their physical activities were significantly impaired or reduced after the cancer diagnosis and related treatment. The results were similar to those of patients with systemic sclerosis, in which physical function had the lowest score of all seven domains (41.90), followed by social health (42.99) [20]. Jensen et al. [21] utilized numerous PROMIS measures in patients newly diagnosed with cancer to evaluate the cancer population-based reference levels, and showed a similar T scores in physical function (44.10), pain interference (52.40), and fatigue (52.20) domains. Most domains in the PROMIS-57 showed an absence of floor and ceiling effects, except for anxiety. The floor effect of the anxiety domain was slightly above the threshold, and the results were similar to those in the general population (28% revealed a floor effect), while no significant ceiling/floor effect was found in the other PROMIS-57 domains [22]. These results were in line with our finding.

Cronbach’s α coefficients of the instrument ranged from 0.89 to 0.95, providing evidence of high internal consistency reliability. The reliability results of the instrument in patients who received a chronic kidney transplant showed that the Cronbach’s α coefficients for each domain ranged from 0.93–0.98, which confirmed our results [23].

The correlations between the item with its domain and that of other domains, together with each domain and the selected FACT-B domains, supported the convergent, discriminant validity, and criterion validity. Measurement invariance was also supported because no DIF was identified for most items regarding age and education. A previous study tested the PROMIS-29 in older adults with multiple chronic diseases, and the researchers reported no significant DIF in this population. Since all PROMIS-29 items were included in the PROMIS-57, the results were not against the present study [24]. Male breast cancer is rare in China, and we failed to recruit male patients with breast cancer in this study. Given that only women were included in this study, male patients must be recruited in a future study to assess the DIF of the PROMIS-57.

The IRT-based analysis could provide more comprehensive item-level psychometric properties when compared with CTT. With few exceptions, most items within the PROMIS-57 were reliable and valid. The unidimensionality structure of most domains was found as hypothesized under the CFA analysis. Tang et al. [23] explored the unidimensionality structure of the PROMIS-57 in patients who received kidney transplantation and found that most of the CFA models fitted well—except the physical function domain, which was slightly higher than the standard boundary value. These results coincided with the original English version [5]. High information of the PROMIS-57 demonstrated that the measure provided power to identify clinical and general population samples, which was consistent with the results of a large sample study of all PROMIS adult profiles [5]. Ideal ICC values further suggested monotonic variation for most items.

This study has several limitations. First, this study only involved patients with breast cancer. Therefore, whether the results can be replicated among patients with other medical conditions should be explored. Second, all participants were recruited from tertiary hospitals, and most of them were middle-aged women receiving surgery and chemotherapy; Third, the cross-cultural validity of the measure was not tested. Thus, future studies should consider patient distribution issues.

## Conclusion

The Chinese version of the PROMIS-57 was a reliable and valid tool for assessing symptoms and functions among patients with breast cancer. Further studies are needed to establish the reliability and validity of the PROMIS-57 in other clinical samples.

## Data Availability

All data presented in this paper are available from the corresponding authors on reasonable request.
